# A Case Report of Dyke-Davidoff-Masson Syndrome in the Philippines: Review of the Literature and a Discussion on Neuroplasticity and Brain Network Connectivity

**DOI:** 10.7759/cureus.80762

**Published:** 2025-03-18

**Authors:** Edren Daeniel G Santos, Geraldine Siena Mariano, Ron Pilotin, Maria Anna Berroya

**Affiliations:** 1 Neurology, St. Luke's Medical Center, Quezon City, PHL; 2 Radiology and Neuroradiology, St. Luke's Medical Center, Quezon City, PHL; 3 Pediatric Neurology, St. Luke's Medical Center, Quezon City, PHL

**Keywords:** brain network, dyke-davidoff-masson syndrome (ddms), epilepsy, neurophysiology. neurology and cognition, neuroplasticity, seizure

## Abstract

Dyke-Davidoff-Masson syndrome is a rare neurologic condition wherein there is characteristic cerebral hemiatrophy that is either congenital or acquired, with a classical presentation of seizures, hemiparesis, and cognitive impairment. Here, we have an unusual case of a young, adult female who sustained a traumatic brain injury in her childhood leading to hemiatrophy but with minimal deficits. This study also summarizes the available data and discusses neuroplasticity and brain network connectivity.

## Introduction

Dyke-Davidoff-Masson syndrome (DDMS) is a rare disease recognized in 1933 by Dyke and colleagues characterized by cerebral hemiatrophy with corresponding ipsilateral compensatory calvarial hypertrophy and paranasal sinus hyperpneumatization [1,2]. Varying etiologies have been reported, including ischemia, trauma, infection, and hemorrhage, to name a few, mainly occurring pre-natally or natally. These causes of injury lead to the reduction of neurotrophic factors necessary for proper brain development [1,3]. Clinically, patients present with hemiparesis, seizures, and cognitive deficits [2]. The relationship between these different etiologies, their symptomatology, and the timing of symptom onset has not yet been fully explored. Furthermore, despite it being recognized for more than nine decades, its exact incidence and prevalence are not known. We present a case from the Philippines, followed by a literature review. We also discuss the implications of DDMS on future research for brain network connectivity and neuroplasticity.

## Case presentation

A 33-year-old right-handed Filipina sought medical consultation for seizure episodes. She was neurotypical, with an unremarkable prenatal, birth, family, and developmental history. At the age of 13, she was involved in a motor vehicle accident. An initial emergency room visit showed no evident craniocerebral trauma on computed tomography scan. She remained asymptomatic until a few weeks after the accident, when her family noticed that she occasionally bumped into objects. She also had episodes of blank stares. She consulted a neurologist at this time, who identified right homonymous hemianopsia on physical examination, later confirmed by visual perimetry.

A cranial magnetic resonance imaging (MRI) with contrast showed an acute to hyperacute infarct involving the left middle cerebral artery (MCA) and posterior cerebral artery (PCA) territories. A subsequent transcranial doppler study suggested <50% stenosis in the left MCA. Angiography of the carotid and vertebral arteries was, however, unremarkable. She was negative for β2-glycoprotein antibodies, lupus anticoagulant, cardiolipin antibodies, and antinuclear antibody. Homocysteine, protein S, and protein C were within normal levels. She was positive for anti-phosphatidylserine IgM and MTHFR mutation (C677T and A1298C mutation). Electroencephalogram reported occasional sharp discharges over the left hemisphere without any areas of focal or generalized slowing. She was managed as a case of pediatric stroke with epilepsy and started on aspirin and carbamazepine. Since this initial set of tests and over the next two decades, she regularly consulted multiple neurologists, remaining compliant with her carbamazepine but not with her aspirin. She rarely had seizure episodes, usually only triggered after long bouts of sleeplessness. She was able to pursue her studies, eventually finishing a master’s degree and becoming a mother.

**Figure 1:**
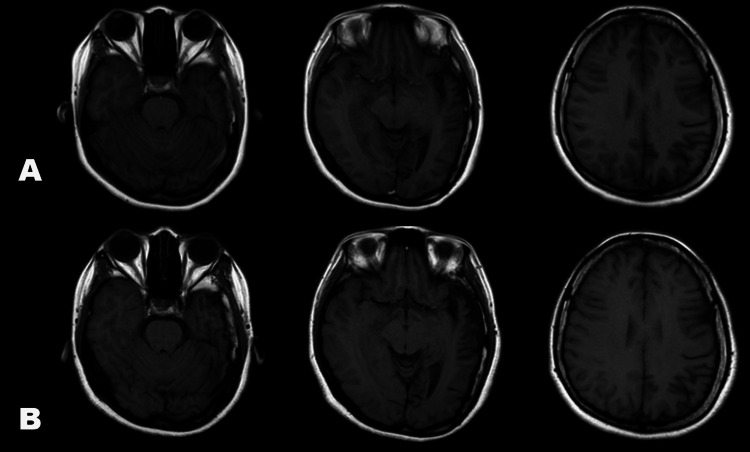
Representative cranial magnetic resonance images (T1 sequences) of the patient. Left cerebral hemiatrophy is apparent in the temporal, occipital, and parietal lobes. Ex vacuo dilatation may be seen in the left occipital area. No significant difference in size were seen between the MRI done in 2018 (Panel A) and 2023 (Panel B).

**Figure 2:**
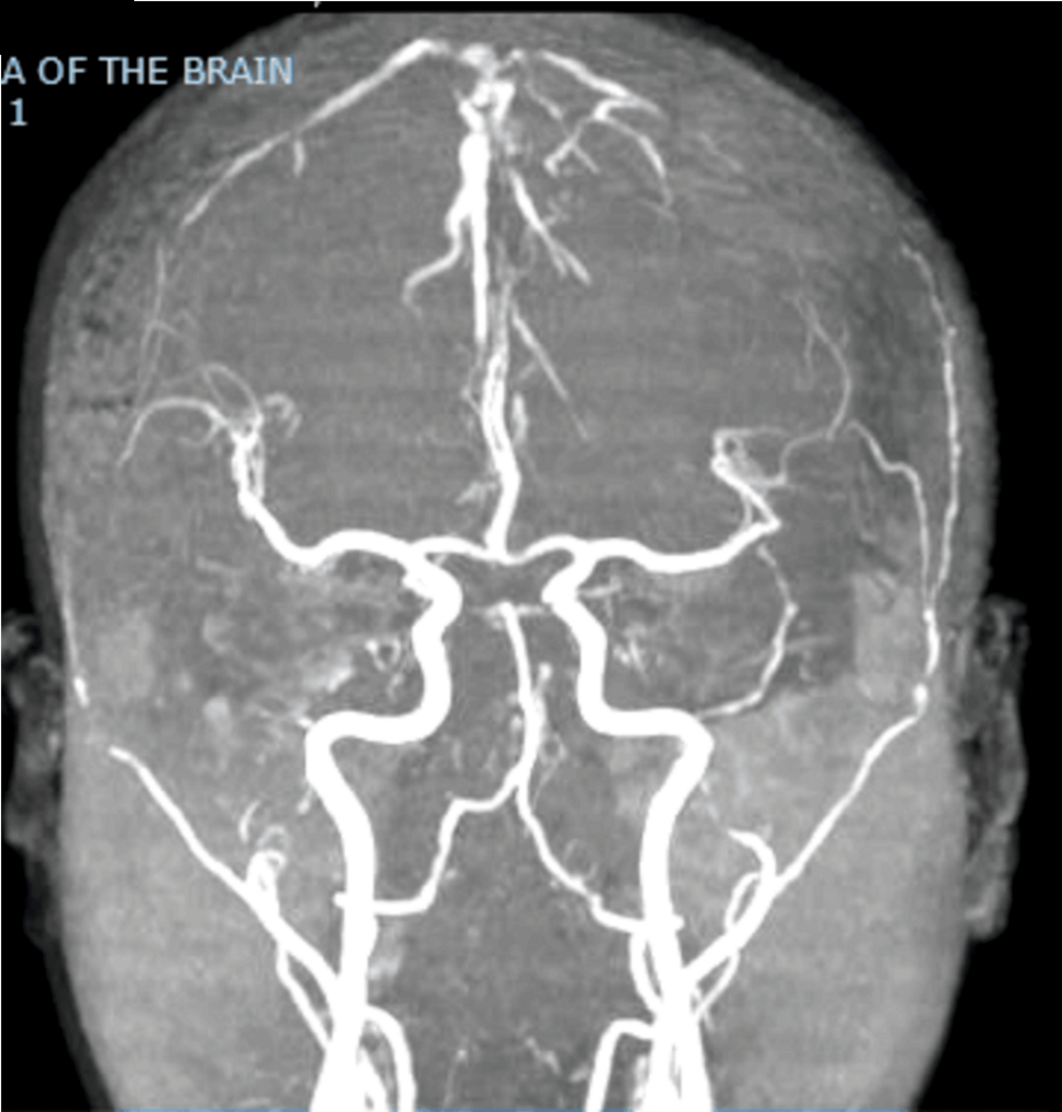
Representative image of the patient's magnetic resonance angiography done in 2023 There were no apparent stenosis, occlusion, or vascular malformations were seen.

By the time she consulted the authors, she still had right homonymous hemianopsia. On cognitive examination, she scored 23/30 on the Montreal Cognitive Assessment - Philippines (MOCA-P), exhibiting difficulties in abstract thinking, memory, and executive functions. The Wechsler Adult Intelligence Scale - IV (WAIS-IV) was administered, revealing a full-scale IQ score of 85, falling within the 'Low Average' range. Areas of difficulty included verbal comprehension, perceptual reasoning, working memory, and processing speed. Both, MOCA-P and WAIS-IV suggest mild cognitive impairment. A repeat contrast cranial MRI was done revealing a stable degree of left cerebral hemiatrophy (Figure 1). Magnetic resonance angiography (MRA) did not reveal any areas of stenosis or occlusion, consistent with the formal catheter angiography done twenty years prior (Figure 2). A repeat electroencephalogram was negative for abnormal slowing and epileptiform discharges.

## Discussion

In Dyke and colleagues' original work in 1933, they described nine patients who presented with hemiparesis and other associated symptoms such as seizures or cognitive disability. This syndrome had characteristic ipsilateral calvarial thickening and sinus dilatation on skull X-rays. On pneumoencephalogram, there was an apparent decrease in calvarial cavity, dilated ipsilateral ventricles, and absent sulci [1]. Since then, additional reports of this syndrome have emerged, now bearing their namesake. In our literature review, we identified a total of 76 studies, including case reports and case series, published between 1972 and 2025, collectively reporting 124 cases of this syndrome.

The mean age of symptom onset is 3.3±0.88 years, though it varies widely, with cases reported as early as birth and as late as 23 years old. Al-Smair and colleagues (2022) reported a case of a female patient who had a stroke at 23 years old, resulting in hemiparesis, and experienced her first seizure at 55 years old. A subsequent cranial MRI showed left cerebral hemiatrophy with ipsilateral calvarial thickening [4]. The mean age of diagnosis is 22.9±3.2 years, but the age of diagnosis is variable, with the majority of cases only being diagnosed at adulthood. The reasons for delay in diagnosis were not specified. There was a case of an 83-year-old male who had been asymptomatic throughout his life until he sustained a fall and cranial imaging revealed unilateral cerebral atrophy [5]. Another noteworthy case is that of a 75-year-old female who was diagnosed post-mortem. This patient had a stroke at 5-years of age, maintaining right-sided hemiparesis without any subsequent seizure or cognitive deficit [6]. Some case series report a male predominance. In a study by Atalar et al, 11 (57.9%) of their 19 patients were male [7]. A smaller series of seven patients had 4 (57.1%) male patients [5]. With our current review of literature, females (51.6%) comprise the majority of the patients.

Many cases had undetermined etiologies, but among those with known causes, the majority were congenital. Other etiologies reported were central nervous system infections, traumatic brain injuries, cerebrovascular disease, neonatal asphyxia, and febrile seizures. There was a report of DDMS probably linked to neuropsychiatric systemic lupus erythematosus by Sordia-Ramirez et al [8]. The contribution of these varying etiologies to hemiatrophy is not yet fully understood. It is postulated that ischemia leads to reduction of neurotrophins important for neural development and plasticity [9].

Despite the varying etiologies, the clinical presentation remains similar. Seizures, hemiparesis, and cognitive and behavioral impairments were the main clinical presentation of majority of the cases. There have been reports of other symptoms, including ataxia, headache, and sensory loss [10-15]. Diagnosis is primarily clinical, but distinct radiologic features can indicate cerebral hemiatrophy.

Tan and colleagues summarized the changes seen in this kind of hemiatrophy syndrome [16]. In addition to typical unilateral cerebral atrophy, bony changes may also be observed, including an adjacent thickened calvarium, pneumosinus dilatans, and hypoplastic middle and anterior cranial fossae [16]. Calvarial changes can help estimate the timing of the injury, as they are mostly observed in patients whose brains have not yet reached adult size, which typically occurs around three years of age [16-18]. In a case series by Atalar, seven (36.8%) of their 19 patients did not show any calvarial changes on their CT or MRI [7]. Interestingly, as their team pointed out, these same set of patients had acquired cerebral injury postnatally in the form of trauma, ischemia, hypoxia, or infection [7]. This can be seen in the patient's MRI, which did not show any bony changes, likely because the patient received her neurologic insult relatively late at the age of 13 years. Other radiologic features include dilatation of the ipsilateral lateral ventricle, sulcal widening, midline shift, basal ganglia hypoplasia, and cerebellar diaschisis and atrophy [16]. These were also not seen in our patient.

There are other differentials pertaining to a patient with cerebral hemiatrophy. These hemiatrophy syndromes can be categorized under either progressive or non-progressive clinical entities. Progressive syndromes are hallmarked by neurodevelopmental regression with an acute to sub-acute presentation that typically will progress and evolve over time [16]. A common example would be Rasmussen's encephalitis, a chronic and progressive disease defined by cerebral hemiatrophy, intractable seizures, cognitive decline, and hemiatrophy [3,16,18]. Non-progressive syndromes are brought about by a single insult to the brain that would produce a relatively stagnant disease. Clinically, a patient with DDMS presents with a non-progressive course, unlike conditions with an underlying inflammatory or neoplastic etiology [16]. Our patient exhibited a non-progressive course both clinically and radiologically. Electrographically, there are no distinguishing features unique to this syndrome. In Demirtas-Tatlidede and colleagues' series of 5 patients, EEG patterns reported were that of focal theta slowing over the affected hemisphere with some isolated spike and wave discharges [13]. There are no established guidelines or formal recommendations for management, but treatment typically involves supportive measures, seizure control, and physical and cognitive rehabilitation. Surgery may be offered for those with refractory seizures, as seen in Shrestha et al, who achieved seizure freedom a year post hemispherectomy [19]. A summary of the reviewed cases may be seen in Table 1.

**Table 1:** Profile of DDMS cases (n=124) DDMS: Dyke-Davidoff-Masson syndrome Cases collected from various case reports and case series from 1972-2025 [2-15,17-79] ^+^Age grouping definitions: Neonate (birth to 4 weeks of life), Infancy (4 weeks of life to 2 years old), Childhood (3 years old to 9 years old), Adolescence (10 years old to 19 years old), Adulthood (>19 years)

Parameter	Number (%)
Age of Symptom Onset^+^	
Neonate	13 (10.5%)
Infancy	37 (29.8%)
Childhood	32 (25.8%)
Adolescence	6 (5%)
Adulthood	1 (0.8%)
Unknown or unspecified	35 (28.2%)
Age of Diagnosis^+^	
Neonate	1 (0.8%)
Infancy	17 (13.7%)
Childhood	10 (8%)
Adolescence	34 (27.4%)
Adulthood	62 (50%)
Unknown or unspecified	0
Sex	
Male	58 (46.8%)
Female	64 (51.6%)
Unknown	2 (1.6%)
Symptomatology	
Seizures	111 (89.5%)
Hemiparesis	81 (65.3%)
Cognitive and Behavioral Changes	56 (45.2%)
Etiology	
Central Nervous System Infection	15 (12.1%)
Congenital	23 (18.5%)
Neonatal Asphyxia	9 (7.3%)
Traumatic Brain Injury	5 (4%)
Cerebrovascular DIseases	6 (4.8%)
Intractable Febrile Seizures	11 (8.9%)
Others	3 (2.4%)
Unknown	52 (41.9%)

In the reviewed literature, the authors identified two reports from the Philippine setting, one case report and one case series, comprising a total of five Filipino patients [20,21]. The patients presented in these Philippine studies also presented with seizures, hemiparesis, and intellectual disability. There were two patients who were siblings but had unknown etiologies. No apparent hereditary pattern was noted on investigation of these two cases. Diestro et al reported a case of a 22-year-old Filipino male who suffered from a central nervous system infection during infancy, which eventually led to right cerebral hemiatrophy with accompanying seizures and left-sided hemiparesis [21]. Fortunately, the patient’s cognitive functions remained relatively intact. He scored a perfect 30 with his mini-mental status examination and was able to finish a bachelor’s degree in education. Similarly, the authors encountered a case with cerebral hemiatrophy with seizures and cognitive deficits, but with good functional outcomes.

**Figure 3:**
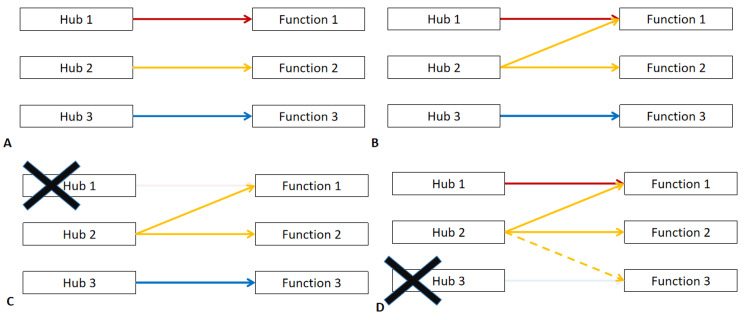
Illustration of functional neural networks and neuroplasticity Panel A illustrates the traditional theory of specific areas of the brain or hubs contribute to a specific function. More recent studies, however, demonstrate that these hubs are interconnected and may contribute to more than one function, such as hub 2 contributing to functions 1 and 2 in panel B. Panel C illustrates an injury to hub 1, and traditionally, one may expect a complete loss of function 1, but the presence of connections in hub 2 prevents this. Panel D illustrates an injury to hub 3, wherein hub 2 was able to establish a new connection preventing complete loss of function 3.

Overview of connectomics, neuroplasticity, and their implications on therapeutics and rehabilitation

spite the severity of neurologic injury and cognitive deficits, the patient has remained functional, not only in performing basic tasks at home but also academically, successfully completing a master’s degree. This is a remarkable demonstration of neuroplasticity, which can be loosely defined as the brain’s ability to adapt in response to various internal and external stimuli, and its correlation with the overall flexibility of brain network connectivity [80,81]. Studies using direct electrical stimulation have supported a homotopic model, in which specific cortical areas function as hubs connected by white matter bundles, facilitating various cerebral functions [82]. Through these same studies, it has been demonstrated that different cortical areas may have multiple functions depending on their connections to other cortical areas. Different cortical-to-cortical and cortical-to-subcortical networks may work in parallel with each other for certain specific functions. Hence, it can be surmised that different networks may adapt and compensate in response to dysfunction of other networks (Figure 3). This has been demonstrated in direct electrical stimulation studies in slow-growing brain tumors, for example, where excision of large tumors seemingly violating Broca’s area or Wernicke’s area failed to produce significant speech defects or language comprehension issues, respectively, post-operatively. Furthermore, the cerebral cortex has been found to demonstrate a greater neuroplasticity potential as opposed to subcortical white matter tracts [80,82]. This highlights the importance of subcortical connectivity and preservation of white matter tracts, which may actually be grossly seen in the patient’s cranial images. Furthermore, the density of network interconnections was found to be directly proportional to the effectiveness of neuromodulation, which is essential for learning [81]. Amongst children and adolescents, this neuroplasticity has been demonstrated through functional neuroimaging, especially after cognitive-based or sensorimotor-based intervention, across age groups who are either neurotypical, neurodevelopmentally delayed, or neurologically injured [83]. This may be brought about through different plasticity mechanisms, such as neuronal and synaptic plasticity. Frank rewiring may also contribute to long-term learning and behavior, but its causality in human models remains incompletely understood [8]

"From a therapeutic perspective, treatments could be designed to promote or accelerate neural and synaptic modulation, as well as reconnectivity, to facilitate proper neural recovery. At a molecular level, one may administer agents that inhibit molecular brakes (e.g. myelin-associated proteins, myelin-associated glycoprotein, extracellular matrix proteins, and axon-guidance molecules) for neuronal growth, many of which did not prove to have any significant clinical benefit on trials [81]. Neuronal stimulation through optogenetic mechanisms, transmagnetic stimulation, direct electrical stimulation, or through task-specific rehabilitation may also promote rewiring [81,82]. Lastly stem-cell transplantation may be a consideration for future interventions [82].

Implications for the patient and future practice

The patient did not receive any formal intervention for addressing her cognitive and motor deficits. However, she has proven more than able to function. One hypothesis for this would be the patient’s exposure to a supportive environment where she was pushed to pursue her hobbies (e.g. dancing) and education even at an early age. This would have provided her with a degree of cognitive and physical rehabilitation, helping to offset her impairments. Although not performed, functional cerebral imaging would have been interesting to conduct for this patient. It would be able to establish hemispheric dominance as well as map out the different functional areas of the brain during cognitive tasks. Nevertheless, with a non-progressive cerebral hemiatrophy, whatever the etiology may be, early identification and control of risk factors as well as early rehabilitation to promote neural recovery and neuroplasticity is important.

Implications for future research

Our case demostrates neuroplasticity and the flexibility of cerebral networking. This, however, is not demonstrated concretely through functional neuroimaging or other neurophysiologic studies. The patient’s case may encourage capable researchers and institutions to study the different adaptive mechanisms to neural injury for similar patients. Furthermore, DDMS remains an understudied disease entity with no clear diagnostic criteria, treatment guidelines, or established prognosis. To date, no well-sized case series or cohort studies have been published that explain the disease course based on severity and the subsequent treatment. Case reports shown in the literature review did not reveal any form of long-term follow up. Furthermore, the genetic underpinnings of congenital cases may also be investigated further. There are still questions on the proper use of anti-seizure medications, long-term outcomes in terms of seizure control and cognitive decline in later age, timing and duration of rehabilitation and their corresponding outcomes, as well as overall survival.

## Conclusions

Dyke-Davidoff-Mason syndrome is a rare neurologic entity whose incidence, exact etiology, pathophysiology, and eventual prognosis have yet to be fully unraveled. An attempt at a comprehensive literature review provided limited insight into treatment and long-term outcomes. Further epidemiologic and clinical research may be done regarding this matter. Nonetheless, this case, to a certain degree, points to the possibility of a hopeful prognosis for other patients with this disease entity. It highlights the importance of utilizing the inherent neuroplastic potential of patients through timely intervention and proper support from their social and physical environment. This paper serves to improve awareness and consolidate available data on this condition, as well as stimulate future research regarding this syndrome.
